# Markers of immune dysregulation in pediatric patients with severe bronchiolitis

**DOI:** 10.70962/jhi.20250133

**Published:** 2025-12-26

**Authors:** Katherine Hickey, Conor Gruber, Sofija Buta-Panov, Jo Hsuan Lee, Guillaume Stoffels, Sandeep Gangadharan, Alfin Vicencio, Dusan Bogunovic

**Affiliations:** 1 Department of Pediatric Critical Care, Mount Sinai Kravis Children’s Hospital, New York, NY, USA; 2 Department of Pediatrics, Mount Sinai Kravis Children’s Hospital, New York, NY, USA; 3 Department of Pediatrics, Center for Genetic Errors of Immunity, Columbia University Irving Medical Center, New York, NY, USA; 4 Department of Population Health Science and Policy, Mount Sinai Medical Center, New York, NY, USA; 5 Department of Pediatric Pulmonology, Mount Sinai Kravis Children’s Hospital, New York, NY, USA

## Abstract

Children with bronchiolitis exhibit a unique immunophenotype. We identify a significant difference in plasmacytoid dendritic cells and eosinophils in hospitalized patients as compared to controls, as well as cytokines involved in airway inflammation that are significantly different in hospitalized patients as compared to controls.

Bronchiolitis is defined as an acute viral illness that affects the lower respiratory system. Despite being a common disease in childhood, bronchiolitis requiring hospitalization is rare. Severe disease is associated with significant morbidity including respiratory failure, resulting in ∼5% of children requiring mechanical ventilation (1). In situ and animal studies suggest that viral infection, in addition to a canonical induction of Th1 immune responses, can lead to activation of Th2 immunity and remodeling of the airway epithelium (2). In pediatrics, clinical data suggest a relationship between severe viral infections and future respiratory morbidity (3). These data suggest that bronchiolitis either has long-term effects on the immune system or that differences in host immunity explain variations in the clinical presentation of disease. The purpose of this study is to map the systemic immune response at an unprecedented depth with a novel approach using proteomic immunophenotyping technologies. This study was designed to be a primer for future studies investigating long-term changes in immunity and personalized therapeutics.

16 patients under the age of three admitted with a diagnosis of bronchiolitis and nine non-viral age-matched controls were included. Patients were excluded if they had chronic disease, an atopic history, prematurity before 36 wk, or a bacterial infection. Patients requiring positive pressure ventilation were considered severe and patients requiring hospitalization but not positive pressure ventilation were considered mild. A summary of clinical features is described in Fig. 1 A. Viral illness was diagnosed using Cepheid CoV-2/Flu/RSV diagnostic PCR or the BIOFIRE Respiratory Panel for 15 of the 16 patients. Blood was drawn in sodium heparin tubes and stored at room temperature.

**Figure 1.**
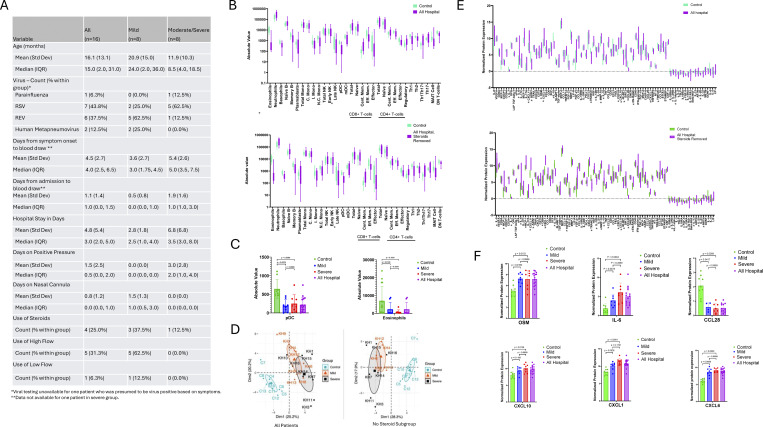
**Clinical and immunophenotyping data for hospitalized patients compared to controls. (A)** Clinical data from the “all hospital,” severe, and mild cohort. Data expressed as frequencies, mean (standard deviation), or mode (interquartile range; IQR). **(B)** Box and whisker plot for immune cell frequency in the hospitalized cohort as compared to healthy control patients (top) and hospitalized cohort as compared to healthy controls with patients receiving any steroids prior to blood draw were removed from the hospitalized cohort (bottom). Hypothesis testing was executed by the non-parametric Mann–Whitney U test. Upper and lower hinges of boxplots correspond to 25th and 75th percentiles and whiskers extend 1.5× IQR from the hinges. **(C)** Bar graph with scatter plot for pDCs in severe patients compared to mild, severe patients compared to control, and mild patients compared to control (left) and eosinophils in severe patients compared to mild, severe patients compared to control, and mild patients compared to control (right). Hypothesis testing was executed by the non-parametric Kruskal–Wallis Test. Upper and lower hinges correspond to 25th and 75th percentiles. Statistical significance is annotated on the graph with P values < 0.05. **(D)** Principle component analysis (PCA) of cytokine expression. Points are colored by sample group classification. Ellipses reflect a 95% confidence interval around the colored group centroid. PCA in severe, mild, and control patients (left). PCA in severe, mild, and control patients with patients receiving any steroids prior to blood draw were removed (right). **(E)** Box and whisker plot for cytokine expression in the hospitalized cohort as compared to healthy control patients (top) and hospitalized cohort as compared to healthy controls with patients receiving any steroids prior to blood draw removed from the hospitalized cohort (bottom). Hypothesis testing was executed by the non-parametric Mann–Whitney U test. Upper and lower hinges of boxplots correspond to 25th and 75th percentiles and whiskers extend 1.5× IQR from the hinges. **(F)** Bar graph with scatter plot for selected cytokine expression in severe patients compared to mild, severe patients compared to control, and mild patients compared to control. Hypothesis testing was executed by the non-parametric Kruskal–Wallis Test. Upper and lower hinges correspond to 25th and 75th percentiles. Statistical significance is annotated on the graph with P values <0.05. Naïve B, naïve B cells; Memory B, memory B cells; Total Mono, total monocytes; C. Mon, classical monocytes; I Mono, intermediate monocytes; N.C. Mono, non-classical monocytes; Total NK, total natural killer cells; Early NK, early natural killer cells; Late NK, late natural killer cells; pDC, plasmacytoid dendritic cells; mDC, mature dendtric cells; Cent. Mem., central memory; Eff. Mem., effector memory; MAIT, mucosal-associated invariant T cells; DN T cells, double-negative T cells.

Written informed consent was provided in compliance with an institutional review board protocol at Mount Sinai Hospital and age-matched controls obtained from discarded blood at Columbia University were IRB exempt. Controls were obtained from children without viral illness and with a normal white blood cell count who required blood drawn for either routine monitoring or follow-up for gastric dysmotility, anemia, or resolved viral illness. Blood was drawn in EDTA tubes at room temperature and processed within 24 h of the blood draw.

Immunophenotyping assays were performed at Mount Sinai Human Immune Monitoring Center. Cytometry of Flight (CyTOF) was performed using Helios mass cytometer (Fluidigm). Data analysis was performed on Cytobank, by biaxial gating of immune populations. For analysis of circulating cytokines, we used the O-link proteomics INFLAMMATION panel, which consists of 92 paired oligonucleotide antibody-labeled probes targeting inflammation-related proteins. Cytometry and cytokine analysis were performed using GraphPad Prism software, version 9. For the primary analysis, Mann–Whitney U tests were used to compare differences in the number of peripheral immune cells and cytokines. The false discovery rate (Q) was used to adjust for multiple comparisons. For our secondary analyses, we performed subgroup comparisons using Kruskal–Wallis testing and linear regression.

32 cell types were evaluated using CyTOF. In hospitalized patients, the immune repertoire was largely intact. However, two immune cell types were significantly decreased in patients compared to controls: plasmacytoid dendritic cells (pDCs) (Q = 0.001) and eosinophils (Q = 0.003) (Fig 1 B). These results posit that circulating pDC activity is different between sick and healthy patients, which is consistent with their crucial role in secreting type I interferons (IFNs), an essential arm of antiviral immunity. Loss of peripheral blood eosinophils may indicate their recruitment to lung tissue, although no tissue samples were collected to assess this directly. In either case, eosinophil depletion may contribute to blurring of Th1 vs. Th2 role in controlling and/or augmenting acute viral diseases.

Our results were consistent when adjusting for steroid use with linear regression. When patients receiving steroids were removed in subgroup analysis, the trend was consistent numerically but lost statistical significance. This is important to note, as steroids can rapidly decrease peripheral eosinophil count, which may have confounded the original analysis. Additional subgroup analysis compared the mild and severe cohort to understand the role of disease severity in immune cell expression. pDCs were significantly decreased in severe (Q = 0.01) and mild patients (Q = 0.02) as compared to control but not severe compared to mild (Fig. 1 C). Finally, we looked at patients with respiratory syncytial virus (RSV) compared to patients with other viral illnesses to explore the role of a particular pathogen in immune cell expression; however, there were no positive discoveries.

For Olink analysis, we hypothesized that severe and mild patients would have discrete cytokine profiles from healthy controls. We first tested this hypothesis with principal component analysis (PCA). While severe and mild patients had some overlaps in their respective cytokine profiles, these cohorts were distinct from that of control patients (Fig. 1 D). PCA was repeated for subgroup analysis without patients receiving steroids yielding similar results (Fig. 1 D). Multiple cytokines were significantly different when hospitalized patients were compared to controls (Fig. 1 E). While there was a pro-inflammatory signature marked primarily by IL-6 (Q = 0.004) elevation, other inflammatory mediators like IL-1 family cytokines and RANKL were downregulated. We also observed increased expression of cytokines related to airway inflammation and mucosal immunity including OSM (Q < 0.001), CXCL1 (Q < 0.001), CXCL6 (Q < 0.001), and CXCL10 (Q = 0.008). When patients with RSV were compared to patients with other viral illnesses, there were no positive discoveries.

Results were similar when patients receiving steroids were removed (Fig. 1 E); however, eight cytokines including IL-α, IL-13, IL-20, IL-22RA1, CCL-28, MMP10, STAMBP, and FGF-5 lost significance in linear regression analysis. Cytokines identified as important drivers of lung inflammation were used for subgroup analysis (Fig. 1 E). These cytokine levels were significantly different among severe patients compared to mild patients and mild compared to control except CXCL1 and CXCL10, in which case there was only a significant difference between severe patients compared to control.

Finally, we aimed to look specifically at cytokines targeted by readily available therapeutics. We performed a subgroup analysis including targets for specific monoclonal antibodies. IL-6 (Q < 0.001) was the only cytokine that was upregulated. Although not statistically significant, IL-6 was notably higher in the severe cohort as compared to the mild cohort, suggesting that a larger analysis may be important for detecting significant differences going forward (Fig. 1 F).

This study demonstrated that hospitalized patients with clinical bronchiolitis have a unique immunologic phenotype as compared to non-viral controls. Enumeration of immune cell subtypes shows significantly decreased pDCs and eosinophils in the peripheral blood of hospitalized patients as compared to controls. pDCs are known for their production of type I IFNs, particularly IFN-α subtypes, which are essential for antiviral immunity and immune response regulation. In fact, genetic deficiency of pDC development via mutation of *IRF7* leads to severe susceptibility to viral infection (4). This finding was consistent even when patients receiving steroids were removed.

Our study is unique in the ability to subgroup patients by disease severity. It is worth noting that while both groups of patients exhibited reduced pDC counts, severe patients had a trend toward higher counts of peripheral pDCs compared to mild. Whether this relative difference represents a cause or effect of increased viral disease severity is uncertain, but several possibilities could explain a pathogenic role. If increased circulating pDCs arise from reduced tissue recruitment, then disease severity may be a function of local pDC activity in affected tissues like the lung. Alternatively, a “Goldilocks zone” of pDC activity may exist for pDC counts in bronchiolitis, where there is sufficient antiviral activity without incurring excess inflammatory damage from type I IFNs. Similarly, eosinophils were decreased peripherally in hospitalized patients as compared to control, consistent with RSV literature reporting lower circulating eosinophils (5), which also may suggest greater eosinophil activity at mucosal sites in viral patients. However, our subgroup analysis suggests that larger sample sizes are needed to explore the effect of steroids and eosinophils.

Our study also provides a unique proteomic analysis to characterize cytokine expression in pediatric bronchiolitis patients. Overall, patients with viral bronchiolitis presented with elevation in multiple cytokine families, particularly in relation to acute phase inflammation, mucosal immunity, and T cell immunity. This signature was consistent across groups, as severe, mild and control patients exhibited unique clusters with PCA analysis.

Our final subgroup analysis focused on cytokine pathways for which monoclonal antibodies or small molecule inhibitors exist that target the corresponding ligand, receptor, or downstream signaling cascade. Among these, IL-6 expression was significantly upregulated in hospitalized patients as compared to controls. Severe patients trended toward higher levels of IL-6 when compared to mild, although larger sample sizes are required to confirm this result. If disease severity is in fact driven by host inflammation, then there may be a role for the use of biologics in severe disease.

Our study offers a novel application of proteomic technologies in the pediatric population. Children with bronchiolitis exhibit a unique immunophenotype with respect to immune cell and cytokine expression. Study limitations include modest sample size, lack of longitudinal data, lack of tissue samples, small differences in sample collection, administration of steroids, and a bias toward hospitalized patients. Nonetheless, these preliminary findings offer an exciting avenue to design future studies looking at a larger cohort of patients longitudinally. Our study supports the continued use of precision medicine in understanding clinical differences in the pediatric bronchiolitis population.

## References

[1] Fujiogi, M., T. Goto, H. Yasunaga, J. Fujishiro, J.M. Mansbach, C.A. Camargo Jr., and K. Hasegawa. 2019. Trends in bronchiolitis hospitalizations in the United States: 2000–2016. Pediatrics. 144:e20192614. 10.1542/peds.2019-2614 31699829 PMC6889950

[2] Rossi, G.A., S. Ballarini, P. Salvati, O. Sacco, and A.A. Colin. 2022. Alarmins and innate lymphoid cells 2 activation: A common pathogenetic link connecting respiratory syncytial virus bronchiolitis and later wheezing/asthma? Pediatr. Allergy Immunol. 33:e13803. 10.1111/pai.13803 35754131

[3] Garcia-Garcia, M.L., C. Calvo, S. Ruiz, F. Pozo, V. Del Pozo, L. Remedios, N. Exposito, A. Tellez, and I. Casas. 2017. Role of viral coinfections in asthma development. PLoS One. 12:e0189083. 10.1371/journal.pone.0189083 29206851 PMC5716580

[4] Sichien, D., C.L. Scott, L. Martens, M. Vanderkerken, S. Van Gassen, M. Plantinga, T. Joeris, S. De Prijck, L. Vanhoutte, M. Vanheerswynghels, . 2016. IRF8 transcription factor controls survival and function of terminally differentiated conventional and plasmacytoid dendritic cells, respectively. Immunity. 45:626–640. 10.1016/j.immuni.2016.08.013 27637148

[5] Sørensen, K.G., K. Øymar, I. Dalen, T. Halvorsen, and I. Bruun Mikalsen. 2023. Blood eosinophils during bronchiolitis: Associations with atopy, asthma and lung function in young adults. Acta Paediatr. 112:820–829. 10.1111/apa.16666 36627486

